# The evolution of scale sensilla in the transition from land to sea in elapid snakes

**DOI:** 10.1098/rsob.160054

**Published:** 2016-06-08

**Authors:** Jenna M. Crowe-Riddell, Edward P. Snelling, Amy P. Watson, Anton Kyuseop Suh, Julian C. Partridge, Kate L. Sanders

**Affiliations:** 1 School of Biological Sciences, University of Adelaide, Adelaide, South Australia 5005, Australia; 2 Brain Function Research Group, School of Physiology, University of the Witwatersrand, Johannesburg, Gauteng 2193, South Africa; 3 School of Animal Biology and Oceans Institute, University of Western Australia, Crawley, Western Australia 6009, Australia

**Keywords:** sea snake, sensilla, mechanoreceptor, hydrodynamic, sensory, elapid

## Abstract

Scale sensilla are small tactile mechanosensory organs located on the head scales of many squamate reptiles (lizards and snakes). In sea snakes and sea kraits (Elapidae: Hydrophiinae), these scale organs are presumptive scale sensilla that purportedly function as both tactile mechanoreceptors and potentially as hydrodynamic receptors capable of sensing the displacement of water. We combined scanning electron microscopy, silicone casting of the skin and quadrate sampling with a phylogenetic analysis to assess morphological variation in sensilla on the postocular head scale(s) across four terrestrial, 13 fully aquatic and two semi-aquatic species of elapids. Substantial variation exists in the overall coverage of sensilla (0.8–6.5%) among the species sampled and is broadly overlapping in aquatic and terrestrial lineages. However, two observations suggest a divergent, possibly hydrodynamic sensory role of sensilla in sea snake and sea krait species. First, scale sensilla are more protruding (dome-shaped) in aquatic species than in their terrestrial counterparts. Second, exceptionally high overall coverage of sensilla is found only in the fully aquatic sea snakes, and this attribute appears to have evolved multiple times within this group. Our quantification of coverage as a proxy for relative ‘sensitivity’ represents the first analysis of the evolution of sensilla in the transition from terrestrial to marine habitats. However, evidence from physiological and behavioural studies is needed to confirm the functional role of scale sensilla in sea snakes and sea kraits.

## 1. Introduction

Evolutionary transitions from terrestrial to aquatic habitats provide important insights into how organismal traits respond to major adaptive shifts. Unfortunately, opportunities to examine such inferences are limited, because many secondarily aquatic taxa lack living, phylogenetically close, terrestrial relatives. An important exception are the front-fanged hydrophiine snakes (Elapidae), which comprise approximately 100 species of Australo-Melanesian terrestrial snakes, 60 species of fully aquatic viviparous sea snakes and eight species of semi-aquatic oviparous sea kraits (*Laticauda*). The whole group is estimated to share a common ancestor dated between 14 and 26 million years ago (Ma); the semi-aquatic sea kraits form the sister lineage to the terrestrial plus viviparous marine species, and the viviparous marine clade diverged independently from within the terrestrial group only 6–8 Ma [1]. Thus, hydrophiines are excellent candidates for studying the evolution of organismal traits resulting from transitions between land and sea.

Our understanding of how selection pressure shapes morphological and physiological evolution in aquatic hydrophiines has advanced in several areas, particularly in traits relating to locomotion [2–5], gas exchange [6–9], diving [10–12] and osmotic balance [13,14]. A number of studies have also sought to understand the evolution of hydrophiine sensory systems associated with the transition to marine life (e.g. hearing [15], vision [16], pressure detection [17] and chemoreception [18]). Nonetheless, the roles of mechanoreception and hydrodynamic reception in the marine environment remain understudied.

Mechanoreception of the external environment is a sensory modality found across diverse taxa. Most terrestrial animals rely on direct touch with solid surfaces. In contrast, the high density and viscosity of water allows many marine organisms to sense the displacement of water using specialized hydrodynamic receptors [19,20]. Hydrodynamic reception allows the detection of water movement from both biotic sources (e.g. prey, predators and mates) and abiotic sources (e.g. turbulence caused by water currents deflected past physical objects) [21]. Strong selection pressure to evolve hydrodynamic reception is suggested by its ubiquitous presence in fish and cephalopods, both of which have a well-developed lateral line system [22–24]. In addition, many secondarily aquatic tetrapods have evolved hydrodynamic receptors, in some cases via exaptation of tactile mechanoreceptors (e.g. the whiskers of pinnipeds [25,26]).

This study examines the putative sensory organs concentrated on the head scales of terrestrial and aquatic elapid snakes. Here, we refer to these organs as ‘scale sensilla’, but they are variously termed ‘sensillae’, ‘corpuscles', ‘tubercles' and ‘papillae’ in the literature [15,27–30]. In terrestrial elapids, scale sensilla are present on the head in large numbers (approx. 6000 per snake) where they function as tactile mechanoreceptors used for sensing the surrounding substrate by direct contact [27,28,30–34]. In aquatic elapids, the function of scale sensilla remains uncertain owing to the hitherto limited number of physiological and morphological studies. Auditory brainstem responses to water movement have been recorded in the sea snake *Hydrophis* (*Lapemis*) *curtus,* but direct extracellular electrophysiological recordings of individual scale sensilla were unsuccessful [15]. A comparative morphological study that included *H. curtus* found markedly more protruding sensillum ultrastructure in aquatic compared with terrestrial snakes [28]. These studies, as well as reports of sea snakes and sea kraits responding to vibrations and pressure changes [17,35], and the limited role of vision for prey capture in some species [16,36], point to the potential significance of scale sensilla for hydrodynamic reception in aquatic elapid snakes. However, the literature on scale sensilla lacks both quantitative (size and coverage) and descriptive (ultrastructure) analysis across terrestrial and aquatic species [37,38], making it difficult to draw comparative conclusions about the function of sensilla.

This study is the first to quantify the traits of scale sensilla in an ecologically and phylogenetically broad sample of snakes, and to analyse these traits within a phylogenetic framework. We begin with a qualitative assessment of the sensillum ultrastructure on the nasal scale, before undertaking a quantitative examination of the numerical density of sensilla, the mean size of individual sensilla and the overall coverage of sensilla on the postocular scale(s) of four terrestrial, 13 fully aquatic and two independently semi-aquatic species of elapids. We discuss our findings in relation to the hypothesis that scale sensilla have been co-opted from a tactile mechanoreceptor in the terrestrial elapids to a hydrodynamic receptor in the sea snakes and sea kraits.

## 2. Material and methods

### 2.1. Specimens

Traits of scale sensilla were examined in 44 individuals from 19 species in the family Elapidae (table 1). Preserved specimens were obtained from the South Australian Museum, the Western Australian Museum and the Field Museum of Natural History, Chicago. Specimens collected from the same locality were used where possible to minimize intraspecific variation over geographical ranges. Only adult male specimens were used to control for the effects of ontogeny and sexual dimorphism (see electronic supplementary material, S1 and table 1, for specimen list and location).

**Table 1.** Taxonomy, ecology and sample size of the elapids analysed in this study.

taxonomy					ecology^a^		sample size	
subfamily	genus	species	synonyms	taxonomic authority	habitat	foraging area	qualitative^b^	quantitative^c^
Hydrophiinae	*Aipysurus*	*duboisii*		Bavay [39]	fully aquatic	varied	1	1
		*fuscus*		Tschudi [40]	fully aquatic	coral reef		1
		*laevis*		Lacépède [41]	fully aquatic	varied		1
		*eydouxii*		Gray [42]	fully aquatic	sandy-bottoms		1
	*Emydocephalus*	*annulatus*		Krefft [43]	fully aquatic	coral reef	1	2
	*Hydrophis*	*curtus*	*Lapemis curtus, Lapemis hardwicki*	Shaw [44]	fully aquatic	varied	1	5
		*cyanocinctus*		Daudin [45]	fully aquatic	varied		3
		*donaldi*		Ukuwela *et al*. [46]	fully aquatic	turbid estuaries/inshore		1
		*major*	*Disteria major*	Shaw [44]	fully aquatic	varied	1	3
		*platurus*	*Pelamis platura*	Linnaeus [47]	fully aquatic	pelagic		4
		*schistosus*	*Enhydrina schistosa*	Daudin [45]	fully aquatic	turbid estuaries/inshore		4
		*stokesii*	*Astrotia stokesii*	Gray [48]	fully aquatic	varied	1	1
		*viperinus*	*Thalassophina viperinia*	Schmidt [49]	fully aquatic	varied		3
	*Hydrelaps*	*darwiniensis*		Boulenger [50]	semi-aquatic	tidal mudflat/mangroves		3
	*Laticauda*	*colubrina*		Laurenti [51]	semi-aquatic	coral reefs/rocky intertidal	1	2
	*Notechis*	*scutatus*		Peters [52]	terrestrial	varied, coastal habitats		3
	*Pseudonaja*	*textilis*		Duméril *et al*. [53]	terrestrial	varied, arid habitats	1	1
	*Vermicella*	*annulata*		Gray [54]	terrestrial	varied, burrowing		1
Elapiniiae	*Naja*	*kaouthia*		Lesson [55]	terrestrial	varied		4
						total	7	44

^a^Summarized from Wilson & Swan [56] and Cogger [57].

^b^Scanning electron microscopy analysis.

^c^Silicone cast analysis.

This paper follows the most recent nomenclature for sea snakes by using *Hydrophis* as the currently accepted genus-level synonym to include species previously in the genera *Pelamis, Enhydrina, Astrotia, Thalassophina, Lapemis* and *Disteira* [58,59]. Taxa are categorized into terrestrial, fully aquatic or semi-aquatic according to field observations [56,57]. The sea snake *Hydrelaps darwiniensis* is phylogenetically nested within the fully aquatic species as sister lineage to *Hydrophis,* but relies on both marine and terrestrial habitats and is therefore grouped here with the other semi-aquatic taxon, *Laticauda*.

### 2.2. Qualitative analysis

**Figure 1.**
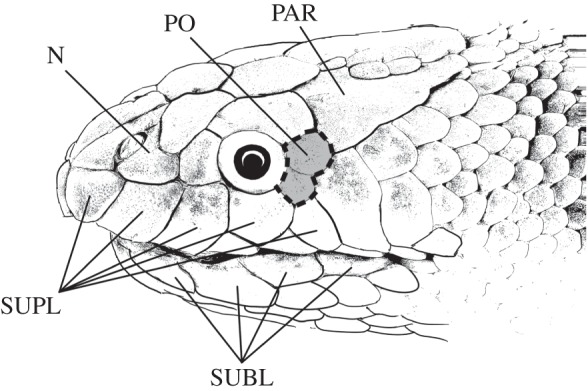
Scale sensilla terminology used in the present study. Nasal (N), supralabials (SUPL), sublabials (SUBL), postoculars (PO) and parietal (PAR). Sampling region for quantitative silicone cast analysis of scale sensilla indicated by dashed line around the postocular scale(s).

High-depth-of-field photographic images of whole snake heads were composed for six representative elapid species comprising one terrestrial species (*n* = 1 individual), four fully aquatic species (*n* = 4 individuals) and one semi-aquatic species (*n* = 1 individual) from the subfamily Hydrophiinae (see electronic supplementary material, S1 and table 2, for details of photography and specimens). In addition, high-magnification images of sensilla ultrastructure on the nasal scale (figure 1) were captured using scanning electron microscopy (SEM) for a subset of elapid taxa, comprising one terrestrial species (*n* = 1 individual), five fully aquatic species (*n* = 5 individuals) and one semi-aquatic species (*n* = 1 individual) from the subfamily Hydrophiinae (table 1)*.* The posterior part of the nasal scale was dissected from museum specimens that had been frozen, fixed in 10% formalin and stored in 100% ethanol. These samples were rinsed in a phosphate-buffered saline solution containing 4% sucrose (pH 7.2), before immersion in a consecutive series of ethanol solutions (70%, 90%, 100%), followed by immersion in hexamethyldisilazane. Samples were then left to air-dry for 5 min before being mounted with an epoxy resin on carbon- or platinum-coated aluminium stubs. The coated samples were then viewed with a high-vacuum, 10 kV SEM (XL30, Philips, Japan). In addition to the nasal scale, the first sublabial, third supralabial, postocular and parietal scales from the sea snakes *Hydrophis major* and *Hydrophis stokesii* were examined directly in environmental SEM (450 Quanta, FEI, USA).

**Table 2.** Morphological parameters quantified from the postocular scale(s) using silicone cast analysis.

parameter	description	units	symbol
number of sensilla	total number of sensilla sampled		*N*_(s)_
total sensilla area	total area of sensilla sampled	mm^2^	*A*_(s)_
total grid cell area	total area of grid cells sampled	mm^2^	*A*_(c)_
numerical density of sensilla	number of sensilla per unit area of postocular scale(s)	mm^−2^	*N*_A(s,c)_
mean sensillum size	mean area of individual sensilla on the postocular scale(s)	μm^2^	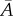 _(s)_
overall coverage of sensilla	total area of sensilla relative to total area of the postocular scale(s)	%	*A*_A(s,c)_

### 2.3. Quantitative analysis

#### 2.3.1. Silicone casting

Quantitative sensilla morphology was examined on the postocular scale(s) (figure 1) of three terrestrial species (*n* = 5 individuals), 13 fully aquatic species (*n* = 30) and two semi-aquatic species (*n* = 5) from the subfamily Hydrophiinae, and one terrestrial species (*n* = 4) from the subfamily Elapiinae (table 1). Following similar methods used for fossilized leaf cuticles [60,61], each snake head was cast in a silicone mould using a two-component, low-viscosity vinylpolysiloxane and black polymer (Pinkysil, Barnes, Australia), which was applied in a series of layers at 30 min intervals. Layering produced casts with an adequate final thickness (approx. 3 mm) and reduced the incidence of bubbles. Fully cured casts (approx. 3–4 h) were peeled off and glued onto cardboard.

#### 2.3.2. Imaging and quadrate sampling

Silicone casts of the postocular scale(s) from each specimen were illuminated with a fluorescent flash and two fibre-optic lights (Studio Dynolite 2000, Dynalite Inc., USA) coupled to a diffuser to reduce specular reflexions from the cast. A high-depth-of-field photographic image was composed for each cast (electronic supplementary material, S1 and table 2), and a 1 mm scale bar was added using imaging software (Adobe Photoshop CS5 Extended, Adobe Systems, USA). Sensilla were quantified from the images using a quadrate sampling method and a script developed with analytical software (MatLabR2015a v. 8.5, MathWorks, USA). The script automatically superimposed approximately 100 grid cells over the postocular scale(s). Sensilla within a systematically random selection of 10 grid cells were then manually identified. Any grid line that crossed a sensillum on the top or right edge of the cell was excluded. The following measurements were then obtained from the images and analysed: total number of sensilla located within the grid cells (*N*_(s)_), total area covered by the sensilla located within the grid cells (*A*_(s)_, mm^2^) and total area of sampled grid cells (*A*_(c)_, mm^2^). Measurements of *A*_(s)_ and *A*_(c)_ were facilitated by the script, which automatically detected the scale bar and provided a pixel-to-area conversion. The numerical density of sensilla (*N*_A(s,c)_, mm^−2^), the mean sensillum size (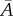
_(s)_, μm^2^) and the overall coverage of sensilla as a percentage (*A*_A(s,c)_, %) on the postocular scale(s) were then calculated for each specimen given *N*_(s)_, *A*_(s)_ and *A*_(c)_ (table 2).

#### 2.3.3. Allometry

To account for the potential effects of head size, *N*_A(s,c)_, 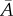
_(s)_ and *A*_A(s,c)_ were scaled against a proxy estimate of head volume (*V*_h_, mm^3^), which was calculated for each specimen as the product of mean head linear measurements (length × width × height). We also tested for the potential effects of *N*_A(s,c)_ on 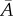
_(s)_, and on *A*_A(s,c)_, because we predicted that the density of organs within the postocular scale(s) would limit the size and coverage of individual sensilla. We used the ‘pic’ function in the ‘ape’ library in R to generate phylogenetic independent contrasts of log_10_-transformed trait data. A linear regression analysis of these data was performed using the ‘lm’ function in the package ‘lme4’ [62–64]. *F*-tests were used to determine whether the exponent for each trait on head size was significantly different from zero. Because 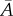
_(s)_ was found to strongly correlate with *N*_A(s,c)_, *A*_A(s,c)_ was used for reconstruction of ancestral states.

### 2.4. Phylogenetic analysis

#### 2.4.1. Sequence data, model selection and data partitioning

DNA sequence data were obtained from GenBank for all 19 elapid lineages. The alignment comprised 3818 base pairs from the mitochondrial genes, cytb (cytochrome *b*), 16S rRNA and 12S rRNA, and the nuclear coding genes, RAG-1 and RAG-2 (recombination reactivating gene 1 and gene 2) and c-mos (oocyte maturation factor). These genes have previously been found to provide sufficient resolution to reconstruct elapid phylogeny and divergence times [58,65–69]. Because DNA sequences were unavailable for *Vermicella annulata* sampled in the morphological analysis, we substituted this species with DNA data from the closely related congener *V. intermedia* in the molecular analysis. Sequences were checked for ambiguities, and alignments were assembled from consensus sequences of forward and reverse reads in Geneious Pro v. 5.1.7 [70]. The appropriate partitioning schemes and best-fit models were selected using Partition Finder v. 1.1.1 [71] under the Bayesian information criterion with branch lengths linked and the greedy search algorithm (table 3).

**Table 3.** Partition schemes and models applied to elapid sequence data and log-transformed traits of sensilla.

partition	locus/trait	model
1	nuclear coding, codon positions 1 + 2	HKY + I + G
2	nuclear coding, codon position 3	HKY + G
3	16S rNA: mitochondrial codon position 1	GTR + I + G
4	mitochondrial codon position 2	GTR + I + G
5	mitochondrial codon position 3	GTR + I + G
6	coverage of sensilla; %	Brownian

#### 2.4.2. Elapid phylogeny and reconstruction of ancestral traits of sensilla

Time-calibrated phylogenies were reconstructed for the concatenated alignment using Bayesian analysis implemented in BEAST v. 1.8.1, which uses a Markov chain Monte Carlo approach to simultaneously estimate topology, divergence times and ancestral character states [72]. The analysis was run with the six-partition scheme and substitution models selected by Partition Finder (table 3). Substitution model parameters were unlinked across partitions, and clock models were linked across partitions. A Yule tree model prior with a uniform distribution was applied. A relaxed clock was used with an uncorrelated and lognormally distributed model of branch rate variation [73]. Because fossils are currently unavailable within Elapidae, two secondary node age priors were obtained from previous molecular dating studies to calibrate divergence times [67]. Prior age distributions were applied to: (i) the split between *Naja* (Elapiinae) and all remaining taxa (Hydrophiinae), using a normal distribution with a mean of 24 million years ago (Ma) and 95% confidence intervals of 15–32 Ma; and (ii) the split between *Laticauda* and all other remaining hydrophiine taxa, using a normal distribution with mean 15 Ma and 95% confidence intervals of 9–22 Ma.

The distributions of ancestral states were estimated for the log-transformed *A*_A(s,c)_. This parameter was treated as a continuous trait under the default Brownian model of character evolution, which allows trait changes to move at a constant and non-directional rate, and is appropriate in the present analysis because traits of sensilla are not yet sufficiently sampled to test alternative (e.g. directional) models of trait evolution [74]. The Markov chain was run for 50 000 000 generations with parameters sampled every 5000 generations. Effective sample sizes for all estimated parameters were assessed using Tracer v. 1.4 [75], and the first 20% of sampled trees were excluded as burn-in. The remaining 8000 trees were used to find the sampled tree with the highest sum of node support values (maximum credibility tree) using Tree Annotator v. 1.7.1 [76]. Tree graphics were adjusted using FigTree v. 1.4.2 [77].

## 3. Results

### 3.1. Qualitative traits of sensilla

**Figure 2.**
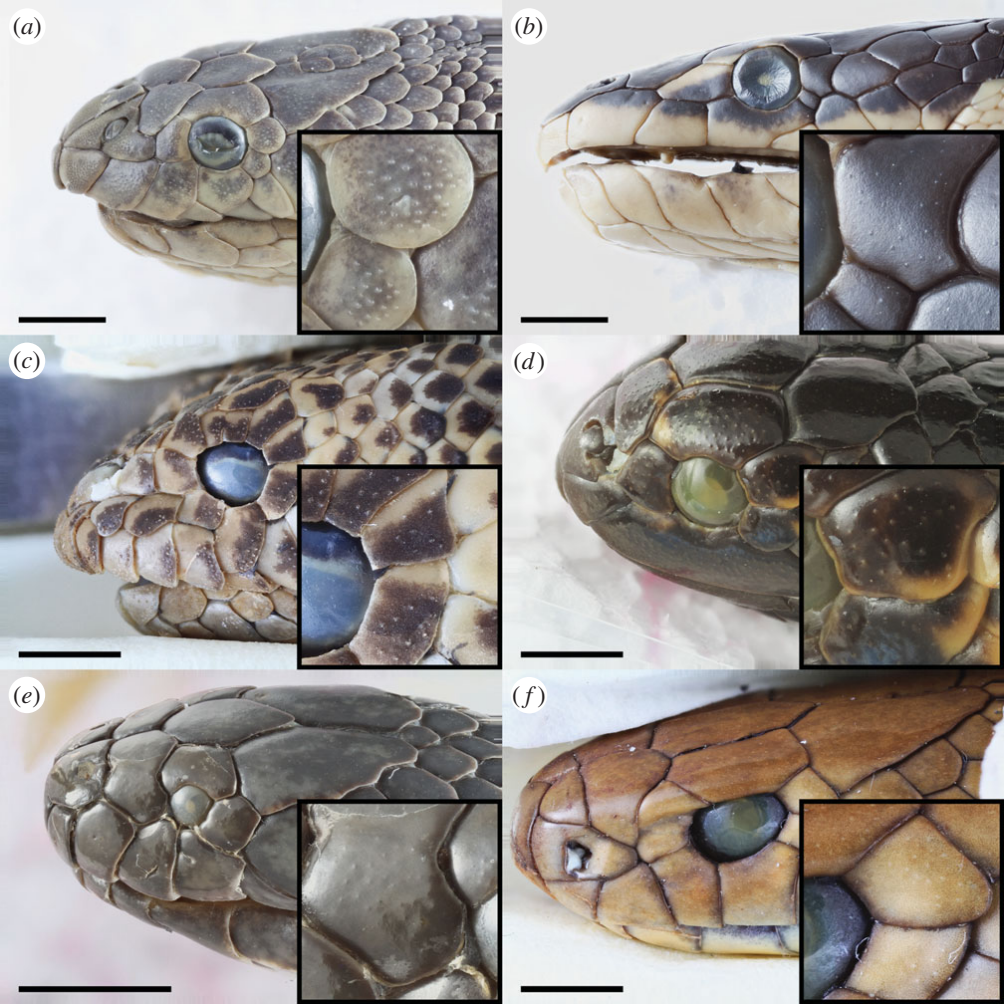
High-depth-of-field photographs of the heads of six elapid species: (*a*) *Hydrophis schistosus*, (*b*) *Hydrophis platurus,* (*c*) *Aipysurus duboisii*, (*d*) *Emydocephalus annulatus,* (*e*) *Hydrelaps darwiniensis* and (*f*) *Pseudonaja textilis.* Species are representative of (*a–d*) fully aquatic, (*e*) semi-aquatic and (*f*) terrestrial ecologies. Insets show sensilla within the postocular scale(s). Scale bar, 3 mm.

**Figure 3.**
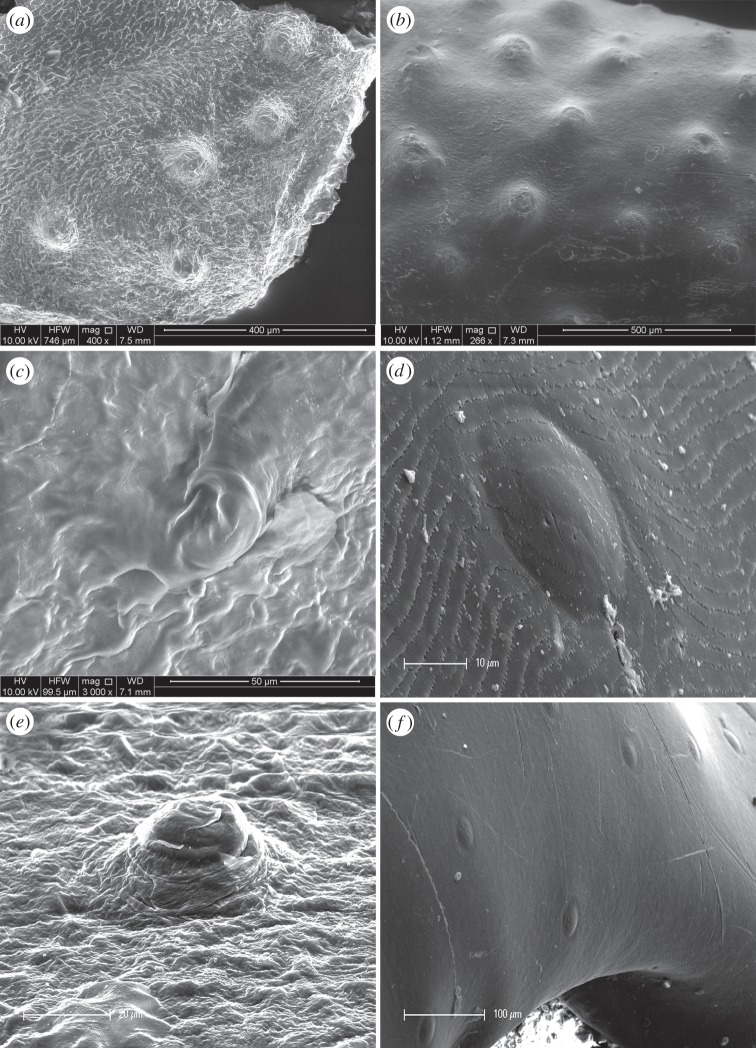
Sensilla viewed under scanning electron microscope on the nasal scale of five species: (*a*) *Aipysurus duboisii*, (*b*) *Hydrophis major*, (*c*) *Laticauda colubrina*, (*d,f*) *Pseudonaja textilis* and (*e*) *Hydrophis curtus.* Species are representative of (*a,b,e*) fully aquatic, (*c*) semi-aquatic and (*d,f*) terrestrial ecologies. Scale bars are indicated for each image (note the variable magnifications).

High-depth-of-field photographic images of elapid heads showed scale sensilla that mostly resembled round bumps protruding from the epidermis (figure 2). Scale sensilla were typically concentrated towards the anterior and lateral sides of the head, and became sparser towards the neck and body. The sensillum ultrastructure imaged under SEM showed that the terrestrial species *Pseudonaja textilis* had numerous flat, elliptical scale sensilla (major axis length approx. 25–30 µm; minor axis length approx. 15–20 µm), whereas the aquatic-associating species had rounder, dome-shaped scale sensilla that protruded prominently from the surrounding epidermis (figure 3). The diameter of sensilla varied greatly between the aquatic species, with the smallest in *Laticauda colubrina* (20 µm), *Hydrophis curtus* (20–30 µm) and *Emydocephalus annulatus* (30 µm), and the largest in *Aipysurus duboisii* (70 µm), *Hydrophis major* (65–75 µm) and *Hydrophis stokesii* (70 µm). In general, the size and shape of sensilla did not vary within an individual.

### 3.2. Quantitative traits of sensilla

#### 3.2.1. Interspecific variation in traits of sensilla

**Figure 4.**
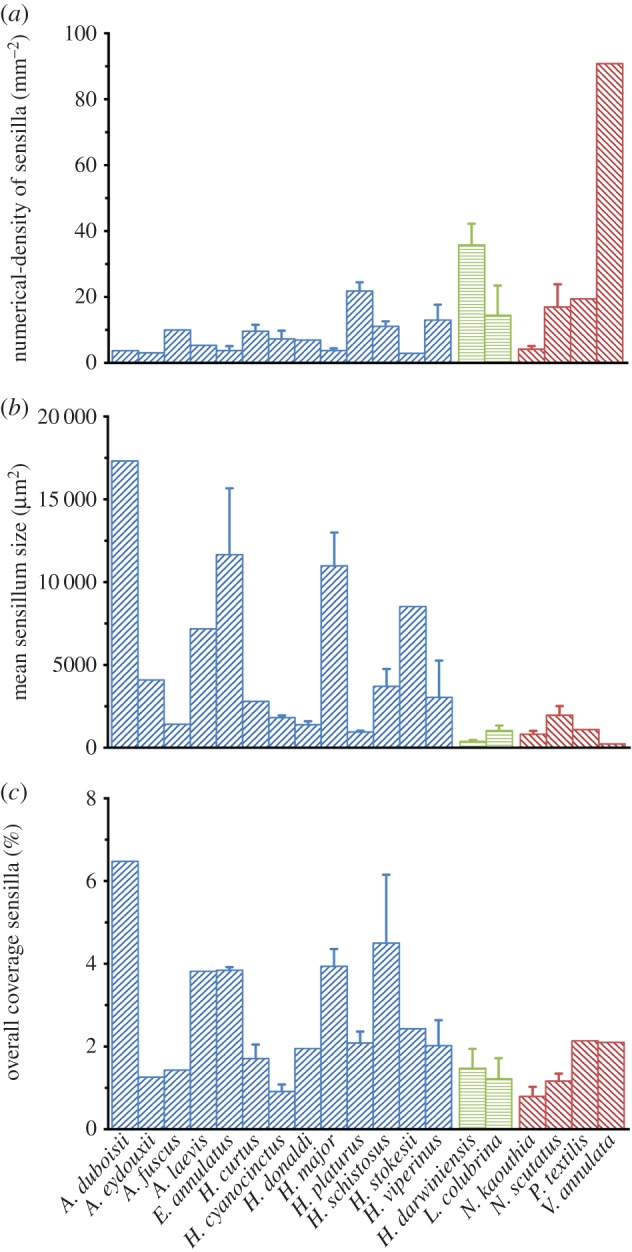
Numerical density of sensilla, mean sensillum size and overall coverage of sensilla quantified from the postocular scale(s) of 13 fully aquatic species (blue), two semi-aquatic species (green) and four terrestrial species (red). Data are means ± s.e.m. calculated from one to six individuals per species (*n* = 44 individuals in total).

Numerical density of sensilla (*N*_A(s,c)_) ranged from 2.8 mm^−2^ in *H. stokesii* to 91 mm^−2^ in *V. annulata* (figure 4). Mean sensillum size (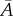
_(s)_) overlapped among aquatic and terrestrial species. Nonetheless, exceptionally large sensilla were found in five fully aquatic sea snakes: *A. duboisii* (17 000 µm^2^), *E. annulatus* (11 700 µm^2^), *H. major* (11 000 µm^2^), *H. stokesii* (8500 µm^2^) and *Aipysurus laevis* (7000 µm^2^). In comparison, the smallest sensilla were found in the following terrestrial and semi-aquatic species: *Notechis scutatus* (800 µm^2^), *Hydrelaps darwiniensis* (400 µm^2^) and *V. annulata* (200 µm^2^). Overall coverage of sensilla (*A*_A(s,c)_) also tended to be higher in fully aquatic species, particularly in the sea snakes, *A. duboisii* (6.5%), *E. annulatus* (3.8%), *A. laevis* (3.8%), *Hydrophis schistosus* (4.4%) and *H. major* (3.9%), compared with the lowest found in the terrestrial *Naja kaouthia* (0.8%). The semi-aquatic species had relatively smaller 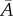
_(s)_ and lower *A*_A(s,c)_ compared with fully aquatic species: *Hydrelaps darwiniensis* (*
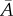
*_(s)_ = 400 µm^2^, *A*_A(s,c)_ = 1.5%) and *Laticauda colubrina* (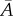
_(s)_ = 1000 µm^2^, *A*_A(s,c)_ = 1.2%).

#### 3.2.2. Allometric effect of head size on traits of sensilla

**Figure 5.**
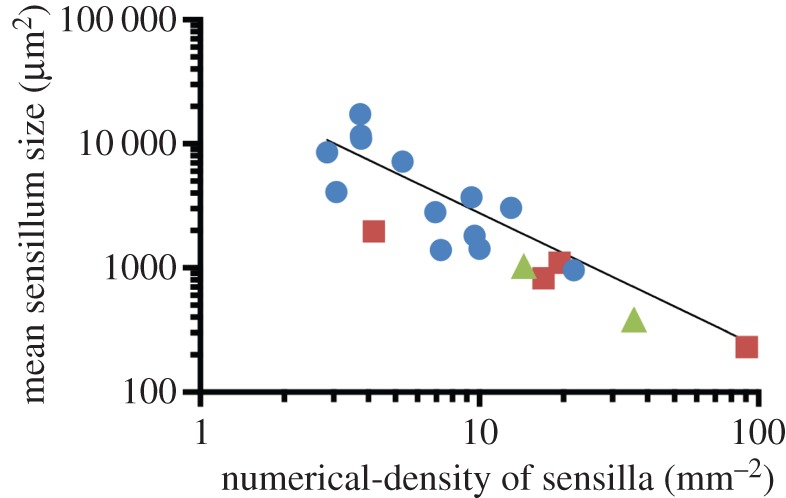
Relationship between mean sensillum size and the numerical density of sensilla quantified from the postocular scale(s) of 13 fully aquatic species (blue circles), two semi-aquatic species (green triangles) and four terrestrial species (red squares). Data are means calculated from one to six individuals per species (*n* = 44 individuals in total).

Regressions of independent contrasts yielded non-significant relationships between traits of sensilla (*N*_A(s,c)_, 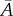
_(s)_ and *A*_A(s,c)_) and head volume (*V*_h_, mm^3^; table 4). Nonetheless, a significant relationship was found between 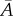
_(s)_ and *N*_A(s,c)_ (*F*_1,16_ = 13.4, *p* = 0.002) with 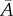
_(s)_ decreasing as *N*_A(s,c)_ increases (figure 5 and table 4). However, *A*_A(s,c)_ was found to be independent of *N*_A(s,c)_ (*F*_1,16_ = 0.0002, *p* = 0.99). Because the terrestrial *V. annulata* is an outlier for head volume, we repeated the regression analyses with this species excluded; this did not change the outcome of our results (not shown).

**Table 4.** Allometric relationship between head volume (*V*_h_) and numerical density of sensilla (*N*_A(s,c)_), mean sensillum size (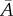
_(s)_) and overall coverage of sensilla (*A*_A(s,c)_) across 19 elapid species. Also shown is the relationship between *N*_A(s,c)_ and 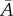
_(s)_, and between *N*_A(s,c)_ and *A*_A(s,c)_. Linear regressions used phylogenetic independent contrasts of mean data calculated from 1–6 individuals per species (*N* = 44 individuals in total). Equations are in the form *y* = *a X*^b^, where *y* is the trait of sensilla, *a* is the coefficient (elevation), *b* is the exponent (slope) and *X* is either *V*_h_ (mm^3^) or *N*_A(s,c)_ (mm^−2^).

traits of sensilla*, y*	*x*	coefficient, *a*	exponent, *b*	95% CI	*r*^2^	d.f.	*F*
*N*_A(s,c)_ (mm^−2^)	*V*_h_	365	−0.13	±0.67	0.04	1,16	0.63
*A*_A(s,c)_ (%)	*V*_h_	1.60	0.11	±0.70	0.02	1,16	0.47
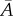 _(s)_ (μm^2^)	*V*_h_	45	0.25	±1.02	0.06	1,16	1.09
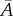 _(s)_	*N*_A(s,c)_	29 800	−1.04	±1.21	0.45	1,16	13.4*
*A*_A(s,c)_	*N*_A(s,c)_	2.80	−0.01	±1.11	1.0 × 10^−5^	1,16	2.0 × 10^−4^

*p = 0.002.

### 3.3. Elapid phylogeny and reconstruction of ancestral coverage of sensilla

**Figure 6.**
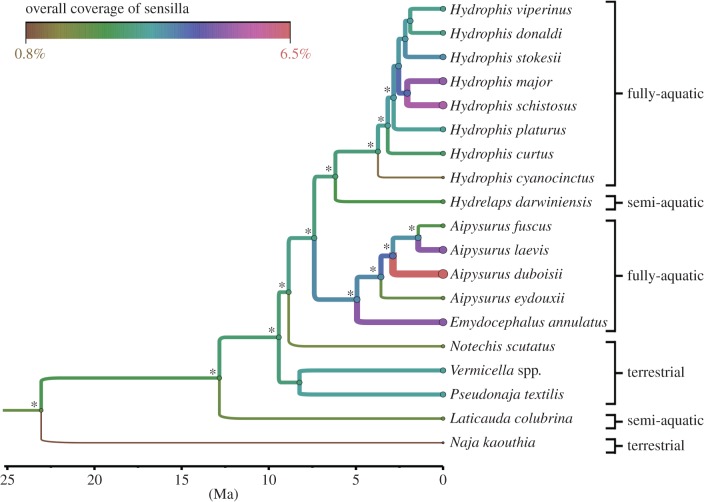
BEAST maximum clade credibility of 19 elapid species with inferred evolution of sensilla coverage. The horizontal axis indicates time scale in millions of years ago. Node posterior probabilities >0.9 are indicated by asterisks. The overall coverage of sensilla (%) is depicted using colour gradient and line weight (warmer colours and thicker branches indicate higher coverage). Because DNA sequences were unavailable for *Vermicella annulata*, DNA data from the closely related congener *V. intermedia* were used in substitute.

The BEAST maximum clade credibility tree (figure 6) is consistent with previous studies in topology, posterior support values and divergence times [1,58,65,67]. The sea snakes are nested within the terrestrial snakes, with *N. scutatus* being their closest terrestrial relative. *Naja kaouthia* (Elapiinae) is sister to all other sampled taxa (Hydrophiinae), and the sea krait *L. colubrina* is the earliest diverging lineage within Hydrophiinae. The most recent common ancestor of the sea snakes is dated at approximately 9 Ma. The two major clades of sea snakes (*Aipysurus* and *Hydrophis*) are recovered as monophyletic sister clades with a most recent common ancestor dated at approximately 7 Ma. As in previous studies, the semi-aquatic *Hydrelaps darwiniensis* is sister to *Hydrophis* and interspecific relationships among the rapidly radiating *Hydrophis* remain largely unresolved [58,78].

The BEAST ancestral state reconstruction for *A*_A(s,c)_ is shown using branch width and colour hues (figure 6). Unusually, high *A*_A(s,c)_ was found only in sea snakes and appears to have evolved multiple times in the fully aquatic *Aipysurus* (*A. duboisii*, 6.5%; *E. annulatus*, 3.8%) and *Hydrophis* (*H. schistosus*, 4.5%; *H. major*, 3.7%) groups. Estimates of ancestral *A*_A(s,c)_ were consistently higher within these fully aquatic clades (1.9–2.8%) compared to within the semi-aquatic and terrestrial lineages (1.5–1.9%). However, *A*_A(s,c)_ was only slightly higher in the common ancestor of sea snakes (2%) than in sampled terrestrial taxa.

## 4. Discussion

Vision, chemoreception and hearing are important senses for terrestrial snakes, but these stimuli have different characteristics underwater, thus altering the selective pressures on sensory systems in elapids that have adapted to aquatic living [79]. It is reasonable to expect that other sensory organs might compensate for the reduced sensory cues in a transition from land to sea. In particular, we hypothesize that the head scale sensilla of sea snakes and sea kraits might function as enhanced tactile mechanoreceptors sensitive to direct contact with solid surfaces, as well as hydrodynamic receptors sensitive to the displacement of water generated by its motion. In this study, we quantify the overall coverage of sensilla as a proxy for relative ‘sensitivity’ in 19 species of elapids encompassing terrestrial, fully aquatic and semi-aquatic ecologies, which we have analysed within a phylogenetic framework.

Our results show substantial variation in the overall coverage of sensilla among elapid species, ranging from 0.8% in the terrestrial cobra *Naja koauthia* to 6.5% in the sea snake *Aipysurus duboisii*. Variation in coverage of sensilla is broadly overlapping in the sampled terrestrial, fully aquatic and semi-aquatic lineages. However, very high overall coverage of sensilla is found in only five (of 13 sampled) fully aquatic sea snakes. In contrast, all of the four terrestrial and two semi-aquatic taxa sampled have consistently lower overall coverage of sensilla. Images under SEM reveal that the sensillum ultrastructure is markedly more protruding (dome-shaped) in the six aquatic hydrophiines that we sampled, in contrast to the flatter sensilla of the single terrestrial species sampled here and the terrestrial species reported in previous SEM studies [27,28,31,80]. These results are discussed below in relation to methodological considerations and the hypothesis that scale sensilla have both a tactile mechanoreceptor function as well as a derived hydrodynamic function in sea snakes and sea kraits.

### 4.1. Allometric effect of head size on traits of sensilla

Allometric scaling showed that the relationship between the traits of sensilla and head volume were all non-significant after accounting for phylogenetic effects (table 4). Nonetheless, there appears to be a trend for a trade-off between mean sensillum size (µm^2^) and numerical density of sensilla (mm^−2^) among the species examined (figure 5). However, overall coverage of sensilla (%) is invariant of numerical density (table 4). Scale organ counts have been estimated in other squamates (e.g. Agamidae, Gekkonidae, Iguanidae, Colubridae, Elapidae, Leptotyphlopidae, Uropeltidae), but these studies do not account for allometric effects, precluding meaningful comparison with our results [27–29,81].

### 4.2. Phylogeny and ancestral reconstruction of the overall coverage of sensilla

BEAST ancestral state reconstruction yielded estimates of overall coverage of sensilla that were only slightly higher for the common ancestor of the fully aquatic sea snakes (2%) than for preceding nodes in the terrestrial elapids (1.5–1.9%; figure 6). *Hydrelaps* and *Laticauda*, which have convergent semi-aquatic habits, also have relatively lower overall coverage, close to values for the terrestrial taxa. Thus, quantitative traits of sensilla do not appear to have undergone dramatic shifts coinciding with transitions to marine habits. However, our analysis reveals independent origins of exceptionally high overall coverage of sensilla in the fully aquatic *Aipysurus* and *Hydrophis* groups, indicating a divergent, possibly hydrodynamic, sensory role in at least some aquatic lineages.

Multiple increases in overall coverage of sensilla in different species of sea snakes may reflect a shifting of receptor sensitivity in response to differing ecologies. The increase in overall coverage of sensilla found in *Hydrophis major* (3.9%) and *Hydrophis schistosus* (4.4%) might reflect increased selection pressure to develop a hydrodynamic sense, because both species specialize on active prey and often hunt in waters with low visibility [82,83]. However, high overall coverage of sensilla in *Emydocephalus annulatus* (3.8%) and *A. duboisii* (6.5%) is less easily explained by their ecology. *Emydocephalus annulatus* usually inhabits clear waters on coral reefs where it specializes on sessile fish eggs [84]. *Aipysurus duboisii* is thought to share similar habitat preferences and foraging habits with closely related *Aipysurus laevis* [82,83], a species that our results indicate has considerably lower overall coverage of sensilla (3.8%) than *A. duboisii*. It is possible that an ecological or behavioural factor that has yet to be discovered in *A. duboisii*, such as nocturnal hunting or mate searching, could explain its unusually higher overall coverage of sensilla compared with all other sampled species.

It is also unclear how sensilla might function in semi-aquatic elapid snakes. The two semi-aquatic lineages sampled here have very different ecologies: *Laticauda* hunts crevice-sheltering prey in clear coral reefs, whereas *Hydrelaps* occupies inshore waters with low visibility but hunts in burrows at low tide [55]. Abrasion during terrestrial locomotion might impose a cost on larger sensilla or higher overall coverage of sensilla. Alternatively, terrestrial life may require particular sensory adaptations to maintain function on land, and evolution of sensilla may be less constrained in fully aquatic snakes. Detailed comparative analysis of the many convergent and divergent ecological specialists within sea snakes and sea kraits [58,83] is needed to shed light on the sensory role of scale sensilla in marine environments.

### 4.3. Comparison of the sensillum ultrastructure

Our qualitative results suggest morphological convergence between scale sensilla on aquatic hydrophiines and the facial organs found in crocodilians and other aquatic snakes. SEM revealed protruding dome-shaped structures in all of the five sea snakes sampled and the single sea krait, whereas comparably flat (two-dimensional) sensilla were observed in the closely related terrestrial species examined here (figure 3) and the eight terrestrial species from the families Colubridae, Xenopeltidae, Cylindrophiidae and Letotyphlopidae examined in previous SEM studies [27,28,31,80]. The dome-shaped ultrastructure is possibly better suited to receiving stimuli from multiple directions, as would be the case for fluid displacement in aquatic habitats [21]. Indeed, the sensillum ultrastructures for the six aquatic hydrophiines are remarkably similar to the dome-shaped papillae of crocodilians, which are sensitive to disturbances on the surface of the water [30,85,86]. Three-dimensional hydrodynamic organs are also found in two non-elapid aquatic snake lineages: the tentacled snake, *Erpeton tentaculatum* (Homalopsidae), and the three species of file snakes in the genus *Acrochordus*. *Erpeton* has large and densely innervated tentacle-like organs on its head that are used for detecting the characteristic escape response of its fish prey [87,88]. In *Acrochordus*, each head and body scale bears dense tufts of fine hair-like protrusions [21,28]. Although the dome-shaped scale sensilla of sea snakes and sea kraits are subtler than the mechanoreceptors of non-elapid aquatic snakes, they might provide greater sensitivity in aquatic habitats compared with the two-dimensional sensilla found in closely related terrestrial species.

### 4.4. Methodological considerations and caveats

There are various methodological hurdles when attempting to compare sensilla across divergent and ecologically diverse taxa. We used a silicone casting technique to make sensilla easily identifiable and minimize taxonomic differences in scale pattern and pigmentation. We also devised a software script to enable quadrate sampling within the postocular scale(s). This approach allowed us to compare traits of sensilla among multiple elapid species, and also generate the first estimate for surface area of sensilla both as the mean sensillum size and overall coverage. Future comparative analyses should aim to expand sampling within species, and include additional taxa (especially terrestrial) to better support statistical testing of the relationships between overall coverage of sensilla and ecological transitions.

Another important caveat is the lack of physiological and behavioural studies supporting a sensory role for scale sensilla, either as a tactile mechanosensory or as a derived hydrodynamic receptor, in sea snakes and sea kraits. Hence, we cannot exclude the possibility of other functional roles. For example, scale sensilla function as electromagnetic receptors used to guide migration or position in the water column [89]. Alternatively, scale sensilla may not be sensory organs at all; higher overall coverage of sensilla might aid in skin shedding, swimming performance, gripping prey/mates or avoiding algae fouling [90,91]. Furthermore, implicit in our interpretations is the assumption that their surface area is a good indicator of their ‘sensitivity’, but this has yet to be empirically tested. Further physiological and behavioural experiments are necessary before we can conclusively link morphological changes in overall coverage of sensilla with a sensory function in sea snakes and sea kraits.

## 5. Conclusion

Our study devised a novel approach to quantify the traits of scale sensilla, which enabled meaningful comparison across a broad sample of elapid snakes. In particular, our estimates of overall coverage of sensilla provided a proxy for putative mechanoreceptor sensitivity and allowed the first analysis of sensilla evolution in the transition from terrestrial to marine habits in snakes. Our results indicate multiple increases in overall coverage of sensilla within the fully aquatic sea snakes, in addition to a more dome-shaped sensillum ultrastructure in fully aquatic and semi-aquatic lineages compared with terrestrial lineages. These findings are consistent with a derived, possibly hydrodynamic, sensory role for scale sensilla in sea snakes and sea kraits, but rigorous testing of this hypothesis will ultimately require behavioural and physiological studies. The novel methodological approach presented here is easily transferable to other reptilian lineages that have undergone adaptive shifts.

## Supplementary Material

Supplementary material

## Data accessibility

Datasets supporting this study have been uploaded as part of the electronic supplementary material.
