# Supplementary material for: The evolution of scale sensilla in the transition from land to sea in elapid snakes
Source: Open Biol. 2016 Jun 8;6(6):160054. doi: 10.1098/rsob.160054 (PMC4929937; doi:10.1098/rsob.160054)
Supplement: Supplementary material [file rsob160054supp1.pdf]

Supplementary material 1

S1 Table 1. List of museum specimens used in this study: Western Australian Museum 'WAM', South Australian Museum 'SAMA', and Field Museum of Natural History, Chicago 'FMNH'.

Genus	Species	Museum	Tag number	Locality
Aipysurus	*duboisii*	WAM	R156216	Exmouth Gulf, Western Australia
	eydouxii	SAMA	R22569	PulauUbin, Singapore
	fuscus	WAM	R129815	Ashmore reef, Western Australia
	laevis	WAM	R174246	Broome, Western Australia
Emydocephalus	*annulatus*	WAM	R129824	Shark Bay, Western Australia
	annulatus	WAM	R165708	Ashmore reef, Western Australia
Hydrelaps	*darwiniensis*	SAMA	R2270D	Darwin, Northern Territory
	darwiniensis	WAM	R22973	Port Headland, Western Australia
	darwiniensis	WAM	R43390	Port Headland, Western Australia
Hydrophis	*curtus*	FMNH	202019	Jahore, West Malaysia
	curtus	FMNH	202021	Jahore, West Malaysia
	curtus	FMNH	202030	Jahore, West Malaysia
	curtus	FMNH	202032	Jahore, West Malaysia
	curtus	FMNH	201910	Jahore, West Malaysia
	cyanocinctus	FMNH	201399	Jahore, West Malaysia
	cyanocinctus	FMNH	201569	Jahore, West Malaysia
	cyanocinctus	FMNH	201572	Jahore, West Malaysia
	donaldi	SAMA	R66274	Weipa, Queensland
	major	WAM	R174252	Broome, Western Australia
	major	WAM	R174253	Broome, Western Australia
	major	WAM	R36550	Carnarvon, Western Australia
	platurus	FMNH	16927	Ecuador
	platurus	FMNH	41591	Piura, Peru
	platurus	FMNH	171674	Costa Rica
	platurus	FMNH	171688	Costa Rica
	schistosus	FMNH	198486	Jahore, West Malaysia
	schistosus	FMNH	206655	Jahore, West Malaysia
	schistosus	FMNH	206657	Jahore, West Malaysia
	schistosus	FMNH	206725	Jahore, West Malaysia
	stokesii	WAM	R174251	Broome, Western Australia
	viperinus	FMNH	201476	Jahore, West Malaysia
	viperinus	FMNH	201578	Jahore, West Malaysia
	viperinus	FMNH	201594	Jahore, West Malaysia
Laticauda	*colubrina*	SAMA	R48012	Babeldaob, Palau
	colubrina	SAMA	R56928	Solomon Islands
Naja	*kaouthia*	SAMA	R63789	Captive, South Australia
	kaouthia	SAMA	R63791	Captive, South Australia
	kaouthia	SAMA	R63792	Captive, South Australia
	kaouthia	SAMA	R63793	Captive, South Australia
Notechis	*scutatus*	SAMA	R18601	Eyre Peninsula, South Australia
	scutatus	SAMA	R25143	Mt Remarkable, South Australia
	scutatus	SAMA	R30505	Williams Is., South Australia
Pseudonaja	*textilis*	SAMA	R18833	Alexandrina, South Australia
Vermicella	*annulata*	SAMA	R13318	Flinders ranges, South Australia

S1 Table 2. High depth of field photographic images of whole-snake heads were composed for six representative hydrophiine species using a series of multi-focus photographs, acquired with a digital DSLR camera (EOS 5D, Canon, Japan) with macro lens (Canon MP-E 65mm, f/2.8 set to magnify 1.4×) and on mount with flashlights (Visionary Digital BK+ Lab Imaging System, Dun, Inc., USA). Resulting images were stacked into a single output using designated imaging software (Zerene Stacker v1.04; Zerene Systems, USA). These photography methods were also utilised for silicone casts of whole-snake heads in Quantitative analysis. Museum specimens from Western Australian Museum 'WAM', South Australian Museum 'SAMA', and Field Museum of Natural History, Chicago 'FMNH'.

**Species**	**Museum**	**Tag number**
Aipysurus duboisii	WAM	R156216
Emydocephalus annulatus	WAM	R165708
Hydrelaps darwiniensis	SAMA	R22973
Hydrophis platurus	FMNH	41951
Hydrophis schistosus	FMNH	201569
Pseudonaja textilis	SAMA	R18833

S1 Table 3. GenBank accession numbers for four mitochondrial and three nuclear genes sampled for 19 sampled elapid taxa in this study. Additional unpublished sequences for sea snakes are shown as 'KLS'.

**Species**	**Locus**						
	**12S**	**16S**	**CMOS**	**cytb**	**ND4**	**RAG1**	**RAG2**
Naja kaouthia	EU624235	JF357948	AY058938	AF217835	EU624209	EU402857	--
Laticauda colubrina	EU547089	EU547138	AY058932	AF217834	EU546998	EU366433	EF144101
Pseudonaja textilis	EU547097	EU547146	EU546914	--	DQ098645	EU546875	--
Vermicella intermedia	EU547104	EU547153	EU546919	--	EF210842	EU546880	--
Notechis scutatus	U96802	--	EU546944	AF217836	EU547034	EU402859	--
Emydocephalus annulatus	EU547136	EU547185	KLS	DQ233942	EU547038	EU546908	--
Aipysurus eydouxii	--	DQ233986	KLS	DQ233910	EF506636	--	--
Aipysurus duboisii	--	DQ233983	KLS	DQ233907	EF506632	--	--
Aipysurus fuscus	--	DQ233987	KLS	DQ233912	EF506634	--	--
Aipysurus laevis	EU547132	DQ233997	KLS	DQ233922	EF506638	EU546906	--
Hydrelaps darwiniensis	EU547133	EU547182	KLS	DQ233948	EU547035	EU546907	--
Hydrophis cyanocinctus	--	DQ234032	FJ587189	DQ233946	FJ593215	FJ587112	--
Hydrophis curtus	EU547134	EU547183	--	DQ233973	EU547036	EU366437	--
Hydrophis platurus	--	DQ234052	--	DQ233978	U49299	--	--
Hydrophis major	--	DQ234018	FJ587186	DQ233937	FJ593209	FJ587108	--
Hydrophis schistosus	--	FJ587210	KLS	KLS	KLS	KLS	--
Hydrophis stokesii	--	DQ234010	FJ587182	DQ233929	FJ593206	FJ587104	--
Hydrophis viperinus		KLS	KLS	KLS	KLS	KLS	
Hydrophis donaldi		KLS	KLS	KLS	KLS	KLS	

Supplementary material 2: Matlab code for morphological calculations

Quadrate sampling of sensilla for size calculations

Table of Contents

Ask the user to select a valid output file 1
Process each image represented in the output 2
Outline sensilla 3
Convert areas to millimetres squared and calculate statistics 4
Calculate area of cell just in case it crosses a region boundary 4
Save current data to file 5
Using regions of interest from previous processing, allows the user to manually outline sensilla in a specified number
of selected subregions

Ask the user to select a valid output file

```
function sensillaAreaCalculation()
% User defined variables
NUM_GRID_CELLS = 100; % change this to alter number of cells in the grid
NUM_CELLS = 3; % change this to alter number of cells to select
prompt = {'Enter number of grid cells:','Enter number of cells to select:'};
defaults = {num2str(NUM_GRID_CELLS), num2str(NUM_CELLS)};
answer = inputdlg(prompt, 'Sensilla Size Calculation', 1, defaults);
NUM_GRID_CELLS = str2num(answer{1});
NUM_CELLS = str2num(answer{2});
[filename, pathname] = uigetfile('output_*.mat', 'Select sensilla data file');
if filename == 0
return;
end
[~,outputName,~] = fileparts(filename);
[~,folderName,~] = fileparts(pathname(1:end-1));
outputFileName = fullfile(pathname, strcat(filename,'.csv'));
outputFolder = fullfile(pathname, outputName);
if ~exist(outputFolder, 'dir')
mkdir(outputFolder);
end
load(fullfile(pathname, filename));
assert(exist('imageData','var') ~= 0, ...
sprintf('"%s" is not a valid sensilla data file.', ...
fullfile(folderName,filename)));
% Load the corresponding calculated values
data = readtable(fullfile(pathname, strcat(outputName,'.csv')));
data = data(:, 1:10); % Only include everything up to area
data.gridHeight = zeros(height(data),1);
data.gridWidth = zeros(height(data),1);
data.cellSize = zeros(height(data),1);
```



```
cellData = data;
cellData(:,:) = [];
warning('off', 'MATLAB:table:RowsAddedExistingVars');
```

Process each image represented in the output

```
numImages = numel(imageData);
numCells = 0;
for i = 1 : numImages
 Load the image and reconstruct the region of interest (ROI)
imData = imageData(i);
image = rgb2gray(imread(imData.imageFilename));
imageHeight = size(image,1);
imageWidth = size(image,2);
roiPoly = imData.roiPolygon;
mask = poly2mask(roiPoly(:,1),roiPoly(:,2), imageHeight, imageWidth);
image(~mask) = 0;
% Crop the image to just the ROI
buffer = 10; % number of border pixels
xMin = max(1, min(roiPoly(:,1)) - buffer);
xMax = min(imageWidth, max(roiPoly(:,1)) + buffer);
yMin = max(1, min(roiPoly(:,2)) - buffer);
yMax = min(imageHeight, max(roiPoly(:,2)) + buffer);
roiRect = [xMin, yMin, xMax-xMin, yMax-yMin];
image = imcrop(image, roiRect);
mask = imcrop(mask, roiRect);
imageHeight = size(image,1);
imageWidth = size(image,2);
 Create a grid of NUM_CELLS cells over the region of interest
aspectRatio = imageWidth/imageHeight;
data.gridHeight(i) = round(sqrt(NUM_GRID_CELLS / aspectRatio));
data.gridWidth(i) = round(aspectRatio * data.gridHeight(i));
xScale = imageWidth / data.gridWidth(i);
yScale = imageHeight / data.gridHeight(i);
mmPerPixelSqrd = data.mmPerPixel(i)^2;
cellSizePixels = (xScale * yScale);
data.cellSize(i) = cellSizePixels * mmPerPixelSqrd;
xGrid = [1, xScale * (1 : data.gridWidth(i))];
yGrid = [1, yScale * (1 : data.gridHeight(i))];
gridLines = [...
ones(size(yGrid')), yGrid', repmat(imageWidth,size(yGrid')), yGrid'; ...
xGrid', ones(size(xGrid')), xGrid',repmat(imageHeight,size(xGrid'))];
image = insertShape(image, 'Line', gridLines, 'Color','white', 'LineWidth',3);
 Ask the user to 'randomly' select cells for processing
for c = 1 : NUM_CELLS
numCells = numCells + 1;
 Manual selection of sensilla
for size calculation
3
cellData(numCells,1:13) = data(i,:);
cla reset;
imshow(image, 'Border','tight', 'InitialMagnification','fit');
hold on;
set(gcf, 'Name', sprintf('%s: Select cell #%d ...', ...
data{i,'ImageName'}{1}, c), 'NumberTitle','off');
pointHandle = impoint();
cellPoint = wait(pointHandle);
delete(pointHandle);
% Work out which cell they selected
cellRect = [0,0,0,0];
for x = 1 : data.gridWidth(i)
if xGrid(x) > cellPoint(1,1)
cellRect(1) = xGrid(x-1);
cellRect(3) = xGrid(x) - xGrid(x-1);
```

```
break;
end
end
for y = 1 : data.gridHeight(i)
if yGrid(y) > cellPoint(1,2)
cellRect(2) = yGrid(y-1);
cellRect(4) = yGrid(y) - yGrid(y-1);
break;
end
end
cellPolygon = bbox2points(cellRect);
% Create new display with image
cla reset;
cellImage = insertShape(image, 'Rectangle', cellRect,...
'Color','red', 'LineWidth',3);
imshow(cellImage, 'Border','tight', 'InitialMagnification','fit');
% Zoom to selected cell
xMin = max(1, min(cellPolygon(:,1)) - 0.5*xScale);
xMax = min(imageWidth, max(cellPolygon(:,1)) + 0.5*xScale);
yMin = max(1, min(cellPolygon(:,2)) - 0.5*yScale);
yMax = min(imageHeight, max(cellPolygon(:,2)) + 0.5*yScale);
xlim([xMin, xMax]);
ylim([yMin, yMax]);
```

Outline sensilla

```
happy = false;
count = 0;
sensilla = {};
while ~happy
choice = questdlg_nonModal('Do you want to ADD any sensilla?', ...
'Add sensilla?', 'Yes','No','No');
happy = strcmp(choice, 'No');
if happy
 Manual selection of sensilla
for size calculation
4
break;
end
% Ask the user to draw an ellipse around the sensilla
sensillaEllipse = imellipse;
sensilla = wait(sensillaEllipse);
if isempty(sensillaEllipse) || isempty(sensilla)
continue;
end
count = count + 1;
sensilla{count} = sensillaEllipse;
% Add text and circle annotations
figureName = sprintf('%s: %d sensilla', ...
data{i,'ImageName'}{1}, count);
set(gcf, 'Name', figureName);
end
% Get area from ellipse objects
sensillaAreas = zeros(numel(sensilla),1);
image = insertShape(image, 'Rectangle', cellRect,...
'Color','green', 'LineWidth',3);
for s = 1 : count
sensilla = getVertices(sensilla{s});
sensillaAreas(s,1) = polyarea(sensilla(:,1), sensilla(:,2));
```

```
poly = reshape(sensilla', 1, numel(sensilla));
image = insertShape(image, 'Polygon', poly, ...
'Color', 'green');
end
imshow(image, 'Border','tight', 'InitialMagnification','fit');
```

Convert areas to millimetres squared and calculate statistics

```
if isempty(sensillaAreas)
sensillaAreas = 0;
end
sensillaAreas = sensillaAreas * mmPerPixelSqrd;
cellData.Number(numCells) = numel(sensilla);
cellData.Minimum(numCells) = min(sensillaAreas);
cellData.Mean(numCells) = mean(sensillaAreas);
cellData.Maximum(numCells) = max(sensillaAreas);
cellData.Sum(numCells) = sum(sensillaAreas);
```

Calculate area of cell just in case it crosses a region boundary

```
cellMask = poly2mask(cellPolygon(:,1), cellPolygon(:,2), ...
```

Manual selection of sensilla
for size calculation
5

```
imageHeight, imageWidth);
cellData.CellArea(numCells) ...
= mmPerPixelSqrd * sum(sum(mask & cellMask));
end % for c = 1 : NUM_CELLS
```

Save current data to file

```
writetable(cellData, outputFileName);
% Save image to output folder
figureName = sprintf('%s_%s_%s_%s_%s_%s.fig', ...
data.Genus{i}, data.species{i}, data.Institution{i}, ...
data.Specimen{i}, data.View{i}, data.Region{i});
savefig(fullfile(outputFolder, figureName));
close gcf;
end
warning('on', 'MATLAB:table:RowsAddedExistingVars');
end
```

Published with MATLAB® R2014

Supplmentary material 3

S3 Table 1. Morphological species data calculated in MatLab using images of specimen whole-head casts.

Genus	Species	Habitat	Sample size	Mean head volume (mm^3)	Sensilla number	Sensilla numerical density (N/mm^-2)	Mean sensillum size (µm^2)	sensilla coverage (%)
Aipysurus	*edouxii*	Fully-aquatic	1	993	6	3.1	4097	1.3
	fuscus	Fully-aquatic	1	2766	10	10.0	1426	1.4
	laevis	Fully-aquatic	1	10979	16	5.3	7188	3.8
	duboisii	Fully-aquatic	1	2570	5	3.7	17314	6.5
Emydocephalus	*annulatus*	Fully-aquatic	2	1609	8	3.8	11665	3.8
Hydrelaps	*darwiniensis*	Semi-aquatic	3	353	13	35.8	386	1.5
Hydrophis	*cyanocinctus*	Fully-aquatic	3	1527	7	7.3	1395	0.9
	curtus	Fully-aquatic	5	2984	15	9.6	1817	1.7
	donaldi	Fully-aquatic	1	410	4	6.9	2805	1.9
	viperinus	Fully-aquatic	3	1712	9	13.0	3046	2.0
	platurus	Fully-aquatic	4	2059	21	21.8	955	2.1
	stokesii	Fully-aquatic	1	7209	5	2.8	8532	2.4
	major	Fully-aquatic	3	5743	7	3.8	10982	3.9
	schistosus	Fully-aquatic	4	1892	8	9.4	6103	4.4
Laticauda	*columbrina*	Semi-aquatic	2	1836	13	14.5	1038	1.2
Naja	*kaouthia*	Terrestrial	4	13739	21	4.2	1968	0.8
Notechis	*scutatus*	Terrestrial	3	4639	15	17.0	825	1.2
Pseudonaja	*textilis*	Terrestrial	1	2309	24	19.4	1102	2.1
Vermicella	*annulata*	Terrestrial	1	116	11	90.8	231	2.1
